# Correction: A distinct p53 target gene set predicts for response to the selective p53-HDM2 inhibitor NVP-CGM097

**DOI:** 10.7554/eLife.19317

**Published:** 2016-11-17

**Authors:** Sébastien Jeay, Swann Gaulis, Stéphane Ferretti, Hans Bitter, Moriko Ito, Thérèse Valat, Masato Murakami, Stephan Ruetz, Daniel A Guthy, Caroline Rynn, Michael R Jensen, Marion Wiesmann, Joerg Kallen, Pascal Furet, François Gessier, Philipp Holzer, Keiichi Masuya, Jens Würthner, Ensar Halilovic, Francesco Hofmann, William R Sellers, Diana Graus Porta

Jeay S, Gaulis S, Ferretti S, Bitter H, Ito M, Valata T, Murakami M, Ruetz S, Guthy DA, Rynn C, Jensen MR, Wiesmann M, Kallen J, Furet P, Gessier F, Holzer P, Masuya K, Würthner J, Halilovic E, Hofmann F, Sellers WR, Graus Porta D. 2015. A distinct p53 target gene set predicts for response to the selective p53–HDM2 inhibitor NVP-CGM097.*eLife* **4**:e06498. doi: 10.7554/eLife.06498.

Published 12, May 2015

We would like to issue the following corrections to our research article entitled, ‘A distinct p53 target gene set predicts for response to the selective p53–HDM2 inhibitor NVP-CGM097’. In the original version of our article, TP53 mutation calls were defined based on the integration of various sources available in 2011, including exon capture sequencing (ECS) data for the majority of cell lines. Using a more recent version of the ECS with full CCLE cell line coverage complemented by the more sensitive Raindance technology, 13 cell lines initially called p53 wt have now been found to be mutant among the 356 cell lines used in the discovery analysis. Similarly, among the 52 cell lines used for validation of the 13-gene signature (external set), 10 cell lines initially called p53 wt have now been found to be p53 mut. A total of 55 PDX models were also used for in vivo validation purposes. Among them, two models initially called p53 wt have been found mutated for p53, and one model initially called p53 mutant has now been confirmed p53 wt.

In Sonkin’s 2015 report, the reannotation of cell lines affected two of our key datasets: 29 cell lines in the 'discovery set' (CCLE panel) (see supplemental file 1C, Sonkin, (2015) and 13 cell lines in the 'validation set' ('external set') (see Table 1, Sonkin, 2015). Table 1 below summarizes the genetic features, including mutation, copy number and mRNA expression published by Sonkin of these cells in both sets.

**Table 1.** Summary of reannotated p53 mut cell lines by Sonkin in the ‘discovery’ and ‘validation sets, based on 'inactivating p53 alterations'.

#	Cell line name	TP53 inactivating mutation(s)	Alternative reads/reference reads	TP53 mRNA (MAS5-150 201746_at)	TP53 CN ratio
‘*Discovery set*’					
1	HPB-ALL	p.C124*	183/21	28	0.55
2	NCI-H1373	p.E339*	8/0	16	0.77
3	SW403	p.E51*	5/0	53	0.68
4	U-937	p.G187_splice	75/0	15	0.84
5	HLF	p.G244A	69/0	252	1.13
6	HEL 92.1.7	p.M133K	22/0	179	0.76
7	BICR 31	p.173_174VR>G	267/1	537	0.9
8	SCaBER	p.R110L	3/0	215	0.75
9	SU-DHL-8	p.R249G, p.Y234N	26/31, 31/27	1804	0.94
10	KMM-1	p.S241F	43/68	46	0.73
11	HCC-78	p.S241F	76/0	201	0.52
12	ES-2	p.S241F	79/0	154	0.97
13	JHOS-2	p.S261_splice	47/51	198	0.65
14	KMS-11			2	0.02
15	COR-L23			795	0.03*
16	MDA-MB-453		6	0.07	
17	MPP 89			1	0.1
18	NCI-H358			7	0.1
19	SK-N-AS			1	0.5
20	MG-63			1	0.7
21	COV644			1	0.74
22	NCI-H2452		1	0.9	
23	PE/CA-PJ49		1	1.01	
24	KP4			3	1.01
25	NCI-H2347		20	0.54	
26	NCI-H2172		24	0.93	
27	L3.3			27	0.72
28	VMRC-RCZ		29	0.52	
29	RERF-LC-AI		31	1.31	
‘*Validation set*’					
1	KASUMI-1	p. R248Q	52/0	265	0.54
2	COLO-818	p. C135R	34/0	257	1.14
3	IGR-37	p. C229fs	110/11	9	0.59
4	HCC202	p. T284fs	35/4	14	0.8
5	EFM-192A	p. F270fs	7/1	10	0.74
6	NCI-H1568	p. H179R	89/1	202	0.82
7	COLO-783	p. P27L	38/0	304	1.05
8	GA-10	p. I232N, p. P152L	94/50, 52/76	493	0.81
9	VMRC-RCW	p. I332_splice	192/68	63	1.65
10	JHH-5	p. PPQH190del	107/41	272	1.03
11	HDLM-2			1	0.94
12	RERF-LC-KJ			25	1.3

*deletion of exons 2,3,4; in red: cells that are selected as p53 mut in our corrected version that were initially considered as p53 wt in our original manuscript.

To reannotate these cell lines included in our original manuscript, Sonkin took into account p53 gene copy number loss and p53 transcript expression on top of p53 mutation status. By doing so, he did not select these cell lines only based on their p53 mutation status, but rather on their p53 functional status. Potential confusion may have arisen based on Sonkin’s title, since his approach was to select cells based on their p53 functional status and not only p53 mutation status.

The purpose of our work was to identify a gene expression signature that could serve as a proxy of p53 functional status. Thus, it is not surprising that Sonkin reported that both approaches yielded similar results. This supports the validity of our approach, namely that we have identified a gene expression signature that is capturing p53 functional status.

In addition, we argue that such a gene expression signature only requires a single technology and hence would be easier to implement in the clinic, since it remains technically challenging, despite the advances in NGS and corresponding gains in sensitivity, to implement Sonkin’s approach. Sonkin’s approach would require multiple technologies to measure p53 gene expression and p53 copy number in addition to sequencing the full-length of p53 for mutation status. Thus, we and others have utilized p53 mutation status (and not p53 expression, copy number, and mutation status) in the clinic, or have explored the use of gene expression-based signatures due to the complexity required in Sonkin’s approach.

It is for these reasons that we developed a gene expression stratification signature and used p53 mutation status as the appropriate benchmark. Importantly, in Sonkin’s report (2015), 13/29 reannotated cell lines in the ‘discovery set’ harbor p53 mutations (shown in red in Table 1). Similarly, 10/12 reannotated cell lines in the ‘validation set’ harbor p53 mutations (shown in red in Table 1). Therefore, we selected these cell lines in both sets that were miscalled for their p53 mutation status only, as it is currently implemented in the clinic, to correct our original manuscript (*i.e.* 13 mutated cells in the ‘discovery set’ and 10 mutated cells in the ‘validation set’ shown in red in Table 1) and not Sonkin’s reannotation (*i.e.* 29 and 12 cell lines in the ‘discovery set’ and ‘validation set’, respectively). Importantly, we continue to show that our gene expression signature with the reannotation is more predictive than p53 mutation status alone.

The corrected version of our manuscript now encompasses the revised analyses of the performance of the 13-gene signature based on the updated p53 mutation calls of the cell lines and patient-derived xenografts (PDX) used in our study. This correction changes the quantitative numbers in the original paper but none of the conclusions are affected. Below are the corrected tables, figures, and text that changed.

The article (text and figures) has been corrected accordingly, and 2 supplementary figures have been added. The original version of the article published on 12 May 2015 is available at https://elifesciences.org/content/4/e06498v2.

The updated Figure 1C, D, E and F reflect the revised p53 mutation calls for the 356 cell lines used in the analysis. In the figure legend for Figure 1 the p-value '3.4 x 10–20' has been corrected to '5.1 x 10–23'.

The corrected Figure 1 is shown below:

**Figure 1.**
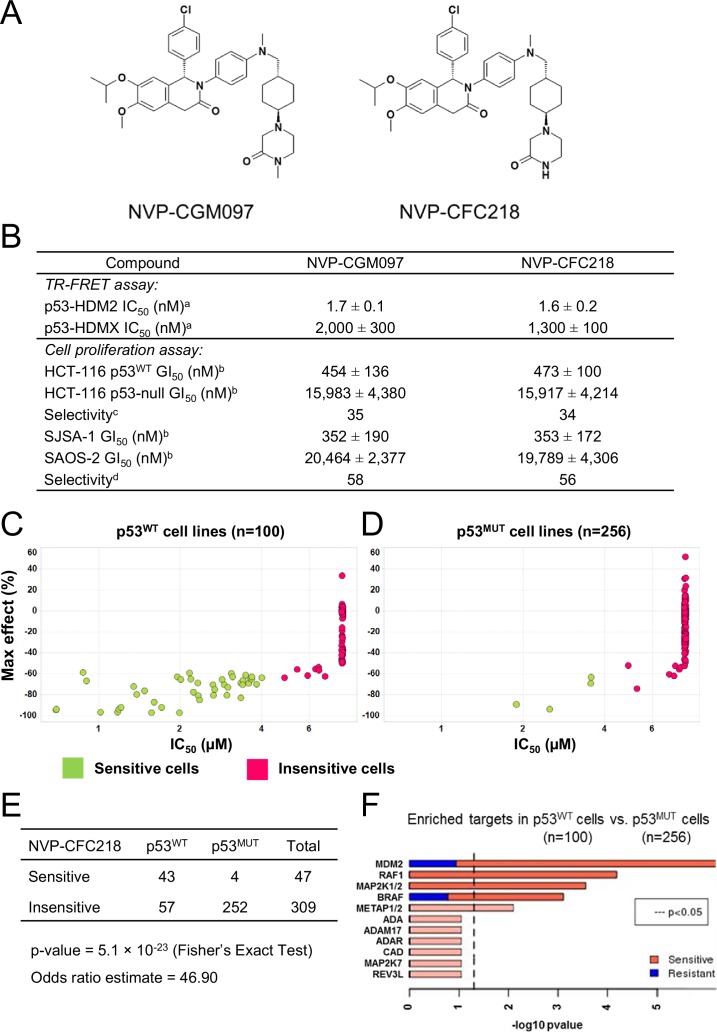
*TP53* wild-type status is necessary but not sufficient to predict sensitivity to NVP-CGM097 and NVP-CFC218. (**A**) Chemical structure of NVP-CGM097 and NVP-CFC218. (**B**) In vitro activity of NVP-CFC218 and NVP-CGM097 in TR-FRET binding assay (^**a**^) and cellular proliferation assay in human cancer cell lines (^**b**^). Data are expressed as concentration causing 50% inhibition and shown as mean ± SD from multiple (n≥8) independent experiments. (^**c**^) Selectivity is determined by the ratio of GI_50_ obtained using the HCT-116 p53-null and the HCT-116 p53^WT^ isogenic pair of cell lines. (^**d**^) Selectivity is determined by the ratio of GI_50_ obtained using SAOS-2 (p53-null) and SJSA-1 (p53^WT^ and *HDM2*-amplified) osteosarcoma pair of cell lines. (C and D) Scatter plot showing IC_50_ values expressed in μM of NVP-CFC218 in cell viability assays of p53 wild-type cell lines (**C**) and p53 mutated cell lines (**D**), colored by their response to NVP-CFC218. The data used to generate these plots, as well as cell line identity are available in Figure 1—source data 1. (**E**) Contingency table indicating the total number of sensitive and insensitive cell lines to NVP-CFC218. The p-value of 5.1 × 10^–23^ shows a significant association between sensitivity to NVP-CFC218 and *TP53* wild-type status. (**F**) Main enriched compound target p-values from the Global Compound Selectivity Analysis. p-values are minus log_10_ transformed. The red color refers to compounds that are more selective in the wild-type p53 (p53^WT^) strata than in the mutated p53 (p53^MUT^) strata. The blue color indicates the reverse profile. Brighter colors indicate which target classes pass the 0.25 FDR cut-off. The length of each red/blue segment corresponds to the proportion of p53^WT^ selective/p53^MUT^ selective compounds in each target class. Figure 1—source data 1.List of cell lines tested for their sensitivity to NVP-CFC218 (n=356).

Figure 2C now provides the ROC curves for the 13-gene signature model and the 215-feature model. We have simplified the p53 mutation status based modeling. Indeed we have not observed any improvements given by the naive Bayes probabilistic algorithm when the p53 mutation status was used in a single feature model. Predictions were identical to the ones given by the mutation status coded into a simple probabilistic model where 0 is the likelihood to be sensitive and 1 the likelihood to be insensitive. Additionally, these probabilities show no variability (a sample will always have the same prediction by its mutation status) and the associated prediction scores take no other values than 0 or 1. It is therefore not informative to use ROC analysis and cross-validated repeats for prediction performance assessment. Thus, the ROC curves for the p53 mutation-based predictions have been removed. Panel 2E shows the updated performance estimates of the p53 mut model. Performance estimates of the 13-gene signature model and 215-feature model are unchanged. The figure legend for Figure 2 remains unchanged.

The corrected Figure 2 is shown below:

**Figure 2.**
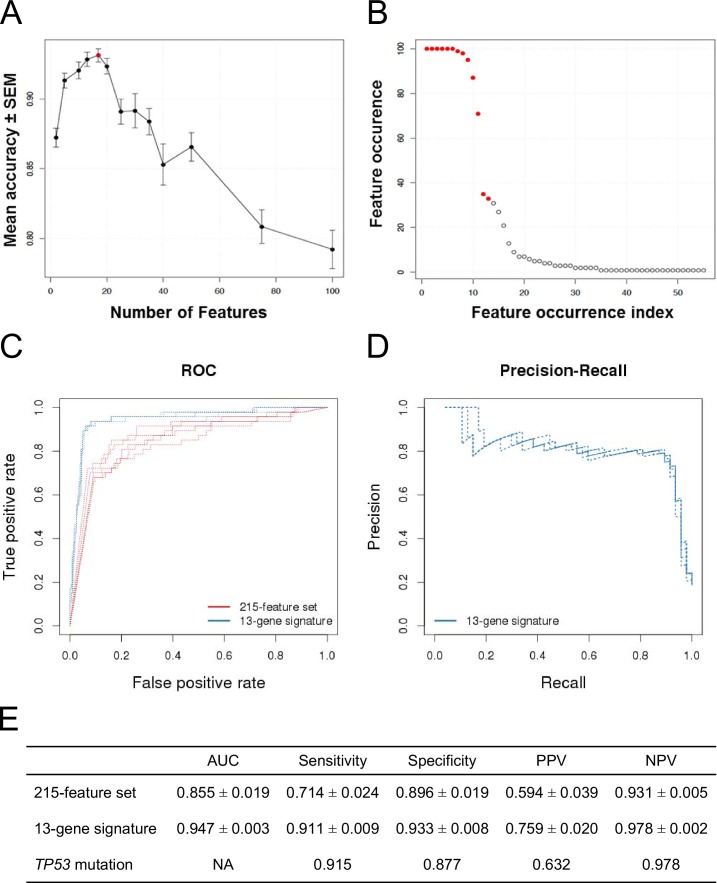
Predictive modeling of NVP-CFC218 chemical sensitivity from CCLE expression data. (**A**) Prediction accuracy estimation by bootstrapping analysis upon increasing feature set size. Estimates are averaged over 20 bootstrapping repeats. The maximum accuracy is observed for 17 features (red dot). Sensitivity and specificity are shown in Figure 2—figure supplement 1. The averaged data used to generate the accuracy, sensitivity and specificity plots are available in Figure 2—source data 1. (**B**) Feature occurrence frequencies of 13 feature selections in 100 bootstrapping repeats. Fifty five features were selected at list once. The 13 most frequently occurring features (red dots) were selected more than 30 times. (**C**) ROC curves for the three predictive models under comparison, *i.e.* the p53 mutation status, the 215-feature and the 13-gene signature models. (**D**) Precision-Recall plot for the 13-gene signature. Five curves are typically shown since cross-validation was repeated 5 times. (**E**) Performance estimates of the three compared predictive models: AUC (Area Under the Curve, from the ROC curve shown in C), Sensitivity (fraction of correctly predicted sensitive cell lines), Specificity (fraction of correctly predicted insensitive cell lines), PPV (positive predicted value, fraction of sensitive cell lines predicted as such) and NPV (negative predictive value, fraction of insensitive cell lines predicted as such). Measures are averaged over the 5 iterations of 5-fold cross-validations.

**Figure 2—figure supplement 2.**
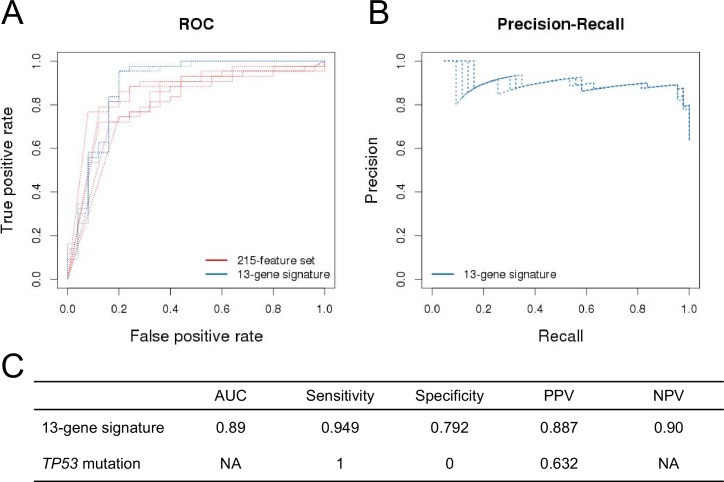
Prediction performance of NVP-CFC218 in p53^WT^ CCLE cell lines. The measures of prediction accuracy are calculated restricted to the wild-type *TP53* subset of CCLE cell lines (n=68). (**A**) ROC curves for the 215-feature set and the 13-gene signature predictive models. (**B**) Precision-Recall plot for the 13-gene signature. Five curves are typically shown since cross-validation was repeated 5 times. (**C**) Performance estimates of the 13-gene signature and *TP53* mutation status as predictive models.

We have added Figure 2—figure supplement 2A,B,C, which is a new figure that was not in the original version of the article. This figure shows a comparison of the performance and prediction accuracies for the '13-gene signature' vs. 'p53 mutation' in the p53 wt cell line population.

We have added Figure 3**—**figure supplement 1A,B,C, which is a new figure that was not in the original version of the article. This figure illustrates the performance of the 13-gene signature in the p53 wt cell line population of the external cell line set for NVP-CGM097 and NVP-CFC218 respectively.

**Figure 3—figure supplement 1.**
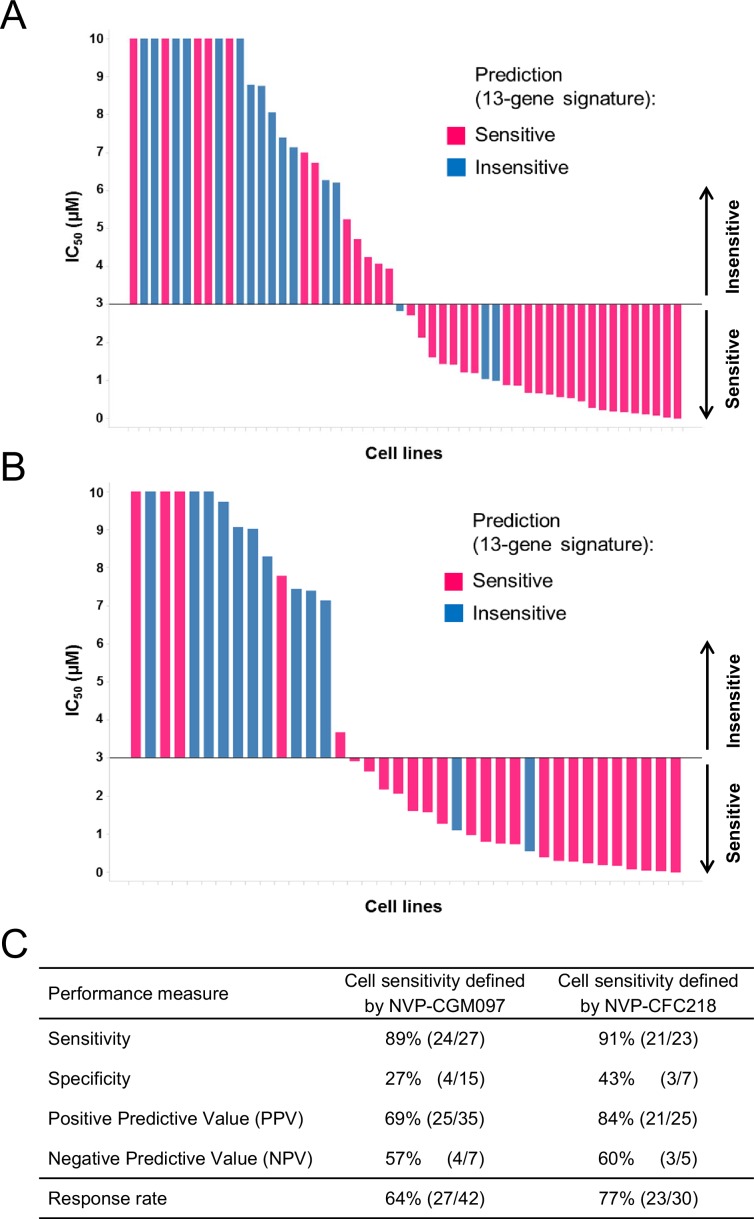
Validation of the predictive 13-gene signature in p53^WT^ cell lines. The same visualization as in Figure 3 is shown restricted to the wild-type p53 cell lines. A total of 42 or 30 p53^WT^ cell lines were tested against NVP-CGM097 (**A**) or NVP-CFC218 (**B**), respectively, in a 3-day proliferation assay. The data used to generate the waterfall plots and cell line details are available in Figure 3—source data 1. The performance values of the 13-gene signature were derived from this experiment, and are shown in (**C**). Figure 3—source data 1.Sensitivity prediction and sensitivity to NVP-CFC218 and NVP-CGM097 of an external set of cell lines (n=52).

Figure 6B and C is updated to reflect the revised p53 mutation calls of the PDX models. In the figure legend for Figure 6 the value 'n=34' has been corrected to 'n=33'.

The corrected Figure 6 is shown below:

**Figure 6.**
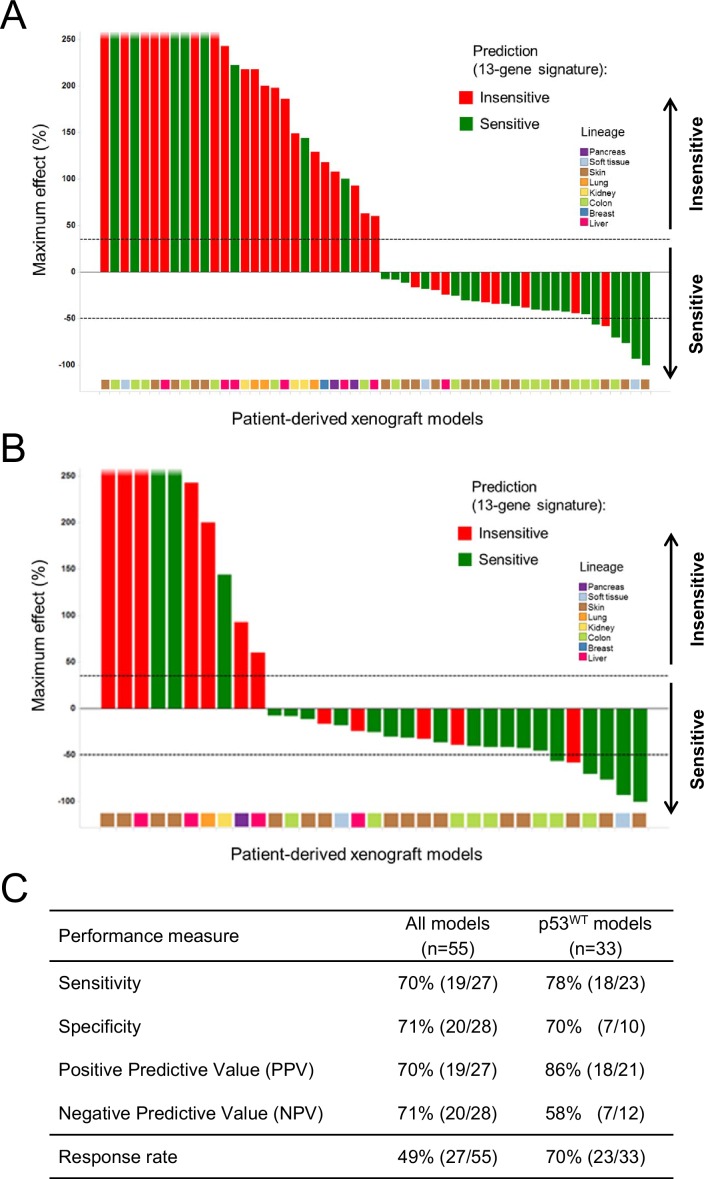
Validation of the predictive 13-gene signature in patient-derived tumor xenografts (PDX). PDX models were established by subcutaneous implantation in nude mice of surgical tumor tissues from treatment-naive cancer patients. 55 PDX models of multiple cancer types were used for the study: melanoma (n = 19), colorectal cancer (n = 17), liposarcoma (n = 3), renal cell carcinoma (n = 3), hepatocellular carcinoma (n = 7), breast cancer (n = 1), pancreatic cancer (n = 2) and lung cancer (n = 3). Tumor-bearing mice (n = 4/dosing group/model) received vehicle or 100 mg/kg of NVP-CGM097 daily for 4 weeks. The response is reported as percentage change in tumor volume at a given day of treatment relative to day 0 (start of treatment). The cut-off used for sensitivity to NVP-CGM097 was based on RECIST adapted for full tumor volume measurement. The sensitivity call was made at the maximum effect time-point during the treatment period. Sensitivity predictions for each PDX model were generated using the 13-gene signature (Figure 6—source data 2). (**A**) Waterfall plot showing the tumor response to NVP-CGM097 for all the PDX models in the study (n = 55), color-coded by the prediction output. (**B**) The same visualization as in (**A**) is shown restricted to the wild-type p53 PDX models (n = 33). The data used to generate the waterfall plots and PDX model details are available in Figure 6—source data 1. The performance values of the 13-gene signature were derived from this experiment, and are shown in (**C**). Figure 6—source data 1.Sensitivity prediction and sensitivity to NVP-CGM097 of a set of in vivo PDX models (n=55).

The updated Figure 1—source data 1 reflects the revised p53 mutation calls for the 356 cell lines used in the analysis.

Title ‘Sensitivity prediction and sensitivity to NVP-CFC218 and NVP-CGM097 of an external set of p53^WT^ cell lines (n=52)’ is now ‘Sensitivity prediction and sensitivity to NVP-CFC218 and NVP-CGM097 of an external set of cell lines (n=52)’. The p53 mut and p53 wt cell lines are indicated in the table.

Figure 6—source data 1 is updated to reflect the revised p53 mutation calls of the PDX models

Corrections to the main text

1. In the Introduction section, we had: ‘The analysis of sensitivity profiles across 355 cell lines led to the confirmation that p53 mutant cancer cells fail to respond to HDM2 inhibitors.’ This has been corrected to ‘The analysis of sensitivity profiles across 356 cell lines led to the confirmation that p53 mutant cancer cells fail to respond to HDM2 inhibitors.’
2. In the Results section, numbers and text have been updated to reflect the revised p53 mutation calls:
  - ‘As expected, based on the mechanism of action of NVP-CFC218, most of the sensitive cell lines harbored wild-type p53 (n=43) and this association was statistically significant by Fisher’s exact test (p-value = 3.4 × 10^-20^) (Figure 1E)’ is now ‘… and this association was statistically significant by Fisher’s exact test (p-value = 5.1 × 10^-23^) (Figure 1E).’
  - ‘Interestingly, among all p53 wild-type cell lines for which compound data were available, 62% scored insensitive to NVP-CFC218 (Figure 1C).’ is now ‘…57% scored insensitive to NVP-CFC218 (Figure 1C).’
  - ‘The *TP53* mutation status was the feature most associated with response to NVP-CFC218 (p-value = 1.17 × 10^-21^), with an odds-ratio of 0.024.’ is now ‘… to NVP-CFC218 (p-value = 2.46 × 10^-26^), with an odds-ratio of 0.013.’
  - ‘We compared ROC (receiver operating characteristic) curves based on the predictions made on the test sets across 5 cross-validation runs for the models based on the 215-feature set, the 13-gene signature and *TP53* mutation status. The AUC for the 13-gene signature was higher than for the other two models: 0.947 *vs*. 0.850 or 0.836 (Figure 2C)’ is now ‘… for the models based on the 215-feature set, the 13-gene signature and the predictions given by *TP53* mutation status (*i.e.* the presence of a *TP53* mutation predicts insensitivity, while a wild-type genotype predicts sensitivity). The AUC for the 13-gene signature was higher than for 215-feature set model: 0.947 *vs*. 0.855 (Figures 2C and E).’
  - ‘Furthermore, the precision–recall curves for the 13-gene signature showed that an 80% true sensitive prediction rate could be achieved while recalling more than 90% of the truly NVP-CFC218-sensitive cell lines (Figure 2D)’ is now ‘… more than 90% of the truly NVP-CFC218-sensitive cell lines (Figure 2D and E).’
  - ‘The class-level performance measures showed that the 13-gene signature provided a substantial improvement in predicting response to NVP-CFC218 in particular when performance was evaluated by Positive Predictive Value (PPV) (Figure 2E): 76% for the 13-gene signature *vs.* 59% for the 215-feature set *vs.* 56% for *TP53* mutation status.’ is now ‘… (Figure 2E): 76% for the 13-gene signature *vs.* 59% for the 215-feature set *vs.* 63% for *TP53* mutation status.’
  - ‘Thus, these results show that the 13-gene signature provides an improvement in response prediction to drug treatment over both the *TP*53 mutation status and the larger signature consisting of 215 significant features.’ is now ‘… and the larger signature consisting of 215 significant features. Moreover, these results suggest that the 13-gene signature has utility in *TP53* wild-type genetic backgrounds.’
  - ‘The predictive value of the 13-gene signature was subsequently tested in a second, independent data set composed of only wild type p53 cell lines of multiple cancer types representative of the lineages included in the first proliferation screen.’ is now ‘The predictive value of the 13-gene signature was subsequently tested in a second, independent data set of cell lines representative of multiple cancer types representative of the lineages included in the first proliferation screen.’
  - ‘As shown in Figure 6A and Figure 6—source data 1, 27/55 human primary xenograft tumor models were predicted to be sensitive to NVP-CGM097, and of these 27 models, 19 were truly sensitive, resulting in a PPV of 70.5%, improving the basal response rate of 49% (Figure 6C).’ is now ‘…resulting in a PPV of 70%, improving the basal response rate of 49% (Figure 6C).’
  - ‘Moreover, 28/55 in vivo models were predicted to be insensitive, and 20/28 were found to indeed be truly insensitive, leading to a significant NPV of 71.5% (Figure 6C).’ is now ‘leading to a significant NPV of 71% (Figure 6C).’
  - ‘As shown in Figure 6B, 22/34 wild-type p53 human PDX models were predicted to be sensitive to NVP-CGM097 and of these, 18 were truly sensitive, resulting in a PPV of 82%, again significantly improving the basal response rate of 65% (Figure 6C). In addition, 12/34 models were predicted to be insensitive to the drug, and 8/12 were found to be truly insensitive, leading to a significant NPV of 66.5% (Figure 6C).’ is now ‘As shown in Figure 6B, 21/33 wild-type p53 human PDX models were predicted to be sensitive to NVP-CGM097 and of these, 18 were truly sensitive, resulting in a PPV of 86%, again significantly improving the basal response rate of 70% (Figure 6C). In addition, 12/33 models were predicted to be insensitive to the drug, and 7/12 were found to be truly insensitive, leading to a significant NPV of 58% (Figure 6C).’
3. Two additional supplementary figures were included, resulting in two additional paragraphs in the Result section:
  - Additionally in a p53 wild-type background (n=68), the 13-gene signature was compared to the p53 mutation model (Figure 2—figure supplement 2). Since in this comparison, the p53 model does not predict any insensitive cell lines, no AUC or NPV can be calculated. The table in Figure 2—figure supplement 2C shows a sensitivity of 94.9%, a specificity of 79.2%, a PPV of 88.7%, and a NPV of 90% for the 13-gene signature. In comparison, p53 mutation model has a sensitivity of 100%, a specificity of 0%, and a PPV of 63.2%. As before, the 13-gene model outperforms the p53 mutation model in terms of PPV (Figure 2—figure supplement 2C).
  - The following sentence ‘These results also indicate the 13-gene signature to be highly predictive even when applied exclusively to the p53 wild-type cell sensitivity.’ Is now ‘We also determined the performance of the 13-gene signature in pre-selected cells based upon the presence of wild-type p53. As shown in Figure 3—figure supplement 1, 35/42 and 25/30 wild-type p53 cell lines were predicted to be sensitive to NVP-CGM097 and NVP-CFC218, respectively, while 25/35 and 21/25 were truly sensitive. PPV values were moderately higher than the basal response rate by 5 to 7%, and the relatively small number of insensitive p53^WT^ cell lines in this external set greatly impaired the specificity and NPV values (Figure 3—figure supplement 1C). Taken together, these results indicate the 13-gene signature to be a better predictive feature than *TP53* mutation status in unselected cell lines. In addition, our results in both the discovery (Figure 2) and the validation sets (Figure 3) suggest the 13-gene signature may improve the response rate in wild-type p53 pre-selected cell lines.’
4. In the Discussion section, numbers and text have been updated to reflect the revised p53 mutation calls:
  - ‘Interestingly, 62% (70/113) of the p53 wild-type cell lines for which compound sensitivity data were available, scored insensitive to NVP-CFC218 (Figure 1C and E).’ is now ‘Interestingly, 57% (57/100) of the p53 wild-type cell lines for which compound sensitivity data were available, scored insensitive to NVP-CFC218 (Figure 1C and E).’
  - ‘The positive predictive value of this 13-gene signature was found to greatly outperform both the p53 mutation feature alone and the 215 significant feature-set (76% *vs.* 56% and 59%, respectively) (Figure 2E).’ is now ‘The positive predictive value of this 13-gene signature was found to outperform both the p53 mutation feature alone and the 215 significant feature-set (76% *vs.* 63% and 59%, respectively) (Figure 2E).’

The original article has been corrected accordingly.

## References

[bib1] Sonkin D (2015). Expression signature based on TP53 target genes doesn't predict response to TP53-MDM2 inhibitor in wild type TP53 tumors. eLife.

