# Supplementary material for: A distinct p53 target gene set predicts for response to the selective p53–HDM2 inhibitor NVP-CGM097
Source: eLife. 2015 May 12;4:e06498. doi: 10.7554/eLife.06498 (PMC4468608; doi:10.7554/eLife.06498)
Supplement: Figure 1—source data 1. — DOI: http://dx.doi.org/10.7554/eLife.06498.004 [file elife-06498-fig1-data1.docx]

**Figure 1-source data 1. List of cell lines tested for their sensitivity to NVP-CFC218 (n=356)**

| Cell Line  Name | Lineage | *TP53*  Mutation  Status | NVP-  CFC218  IC_50_ (µM) | NVP-  CFC218  Amax (%) | NVP-  CFC218  sensitivity  call |
| --- | --- | --- | --- | --- | --- |
| MOLM-13 | haem._and_lymphoid_tissue | WT | 0.69 | -94.52 | sensitive |
| BDCM | haem._and_lymphoid_tissue | WT | 0.70 | -93.74 | sensitive |
| NCI-H1666 | lung | WT | 0.88 | -58.51 | sensitive |
| COLO-679 | skin | WT | 0.91 | -66.48 | sensitive |
| SUP-M2 | haem._and_lymphoid_tissue | WT | 1.01 | -96.77 | sensitive |
| NALM-6 | haem._and_lymphoid_tissue | WT | 1.16 | -96.38 | sensitive |
| DOHH-2 | haem._and_lymphoid_tissue | WT | 1.18 | -93.70 | sensitive |
| KP-N-SI9s | autonomic_ganglia | WT | 1.20 | -92.10 | sensitive |
| CHP-212 | autonomic_ganglia | WT | 1.36 | -71.98 | sensitive |
| KE-97 | stomach | WT | 1.38 | -79.42 | sensitive |
| WM-266-4 | skin | WT | 1.46 | -75.96 | sensitive |
| 697 | haem._and_lymphoid_tissue | WT | 1.59 | -96.96 | sensitive |
| G-401 | soft_tissue | WT | 1.60 | -87.03 | sensitive |
| JHUEM-2 | endometrium | WT | 1.73 | -91.79 | sensitive |
| **NCI-H2122** | **lung** | **MUT** | **1.90** | **-89.07** | **sensitive** |
| Hep G2 | liver | WT | 1.95 | -62.73 | sensitive |
| AMO-1 | haem._and_lymphoid_tissue | WT | 2.01 | -96.94 | sensitive |
| ACHN | kidney | WT | 2.03 | -65.30 | sensitive |
| A-204 | soft_tissue | WT | 2.18 | -58.99 | sensitive |
| KS-1 | central_nervous_system | WT | 2.20 | -64.80 | sensitive |
| A2780 | ovary | WT | 2.24 | -77.43 | sensitive |
| G-402 | soft_tissue | WT | 2.31 | -80.69 | sensitive |
| DK-MG | central_nervous_system | WT | 2.32 | -68.71 | sensitive |
| OCI-AML2 | haem._and_lymphoid_tissue | WT | 2.38 | -84.70 | sensitive |
| **P12-ICHIKAWA** | **haem._and_lymphoid_tissue** | **MUT** | **2.50** | **-93.77** | **sensitive** |
| Reh | haem._and_lymphoid_tissue | WT | 2.63 | -80.40 | sensitive |
| A-375 | skin | WT | 2.65 | -70.39 | sensitive |
| LU99 | lung | WT | 2.71 | -74.84 | sensitive |
| H4 | central_nervous_system | WT | 2.84 | -65.09 | sensitive |
| TE 617.T | soft_tissue | WT | 2.91 | -70.15 | sensitive |
| KYM-1 | soft_tissue | WT | 2.95 | -80.17 | sensitive |
| 769-P | kidney | WT | 3.01 | -72.79 | sensitive |
| Hey-A8 | ovary | WT | 3.12 | -59.27 | sensitive |
| G-361 | skin | WT | 3.16 | -62.79 | sensitive |
| NCI-H2052 | pleura | WT | 3.36 | -68.74 | sensitive |
| HCT 116 | large_intestine | WT | 3.38 | -66.04 | sensitive |
| GRANTA-519 | haem._and_lymphoid_tissue | WT | 3.38 | -70.32 | sensitive |
| WM-115 | skin | WT | 3.38 | -82.69 | sensitive |
| GP2d | large_intestine | WT | 3.45 | -61.10 | sensitive |
| **KMBC-2** | **urinary_tract** | **MUT** | **3.55** | **-69.03** | **sensitive** |
| K029AX | skin | WT | 3.61 | -68.89 | sensitive |
| **Hs 294T** | **skin** | **MUT** | **3.61** | **-63.14** | **sensitive** |
| Caki-2 | kidney | WT | 3.63 | -64.31 | sensitive |
| JVM-3 | haem._and_lymphoid_tissue | WT | 3.64 | -65.00 | sensitive |
| Hs 695T | skin | WT | 3.65 | -62.89 | sensitive |
| HT-144 | skin | WT | 3.86 | -69.73 | sensitive |
| DBTRG-05MG | central_nervous_system | WT | 4.00 | -63.69 | sensitive |
| HCC1500 | breast | WT | 4.89 | -63.62 | insensitive |
| SW 1353 | bone | MUT | 4.91 | -52.12 | insensitive |
| MC116 | haem._and_lymphoid_tissue | MUT | 5.21 | -73.96 | insensitive |
| TOV-21G | ovary | WT | 5.41 | -55.82 | insensitive |
| COV434 | ovary | WT | 5.89 | -61.47 | insensitive |
| NCI-H1944 | lung | WT | 6.39 | -55.40 | insensitive |
| Hs 936.T | skin | WT | 6.44 | -53.45 | insensitive |
| RKO | large_intestine | WT | 6.44 | -56.51 | insensitive |
| KU-19-19 | urinary_tract | WT | 6.89 | -62.34 | insensitive |
| MFE-280 | endometrium | MUT | 6.92 | -60.45 | insensitive |
| LP-1 | haem._and_lymphoid_tissue | MUT | 7.24 | -61.82 | insensitive |
| HGC-27 | stomach | MUT | 7.41 | -52.41 | insensitive |
| MDA-MB-415 | breast | MUT | 7.62 | -55.83 | insensitive |
| Loucy | haem._and_lymphoid_tissue | MUT | 7.62 | -53.62 | insensitive |
| HEC-151 | endometrium | MUT | 7.91 | -52.18 | insensitive |
| HCC4006 | lung | MUT | 8.00 | -3.10 | insensitive |
| SHP-77 | lung | MUT | 8.00 | -4.02 | insensitive |
| MG-63 | bone | WT | 8.00 | -26.13 | insensitive |
| SW1417 | large_intestine | MUT | 8.00 | 7.38 | insensitive |
| L3.3 | pancreas | WT | 8.00 | -23.35 | insensitive |
| COV644 | ovary | WT | 8.00 | -2.94 | insensitive |
| NCI-H1373 | lung | MUT | 8.00 | -0.95 | insensitive |
| BHY | upper_aerodigestive_tract | MUT | 8.00 | 1.51 | insensitive |
| NCI-H520 | lung | MUT | 8.00 | -1.85 | insensitive |
| U-937 | haem._and_lymphoid_tissue | MUT | 8.00 | -25.04 | insensitive |
| NCI-H2172 | lung | WT | 8.00 | -28.81 | insensitive |
| KP4 | pancreas | WT | 8.00 | 0.41 | insensitive |
| PE/CA-PJ49 | upper_aerodigestive_tract | WT | 8.00 | -24.87 | insensitive |
| RERF-LC-AI | lung | WT | 8.00 | 1.17 | insensitive |
| HEC-50B | endometrium | MUT | 8.00 | -23.10 | insensitive |
| HCC-78 | lung | MUT | 8.00 | 0.79 | insensitive |
| L-363 | haem._and_lymphoid_tissue | MUT | 8.00 | 1.09 | insensitive |
| NCI-H1573 | lung | MUT | 8.00 | 7.38 | insensitive |
| COV318 | ovary | MUT | 8.00 | 5.06 | insensitive |
| NCI-H1435 | lung | MUT | 8.00 | 1.81 | insensitive |
| CFPAC-1 | pancreas | MUT | 8.00 | -38.08 | insensitive |
| KP-2 | pancreas | MUT | 8.00 | -23.35 | insensitive |
| NCI-H650 | lung | MUT | 8.00 | -38.20 | insensitive |
| FU97 | stomach | MUT | 8.00 | -1.04 | insensitive |
| SNU-182 | liver | MUT | 8.00 | 4.52 | insensitive |
| HEL 92.1.7 | haem._and_lymphoid_tissue | MUT | 8.00 | 5.00 | insensitive |
| GCIY | stomach | MUT | 8.00 | -4.41 | insensitive |
| EFM-19 | breast | MUT | 8.00 | -9.07 | insensitive |
| NCI-H889 | lung | MUT | 8.00 | 3.61 | insensitive |
| OVCAR-4 | ovary | MUT | 8.00 | 3.62 | insensitive |
| SBC-5 | lung | MUT | 8.00 | -1.36 | insensitive |
| MOG-G-CCM | central_nervous_system | MUT | 8.00 | -5.90 | insensitive |
| PSN1 | pancreas | MUT | 8.00 | -6.25 | insensitive |
| HCC-56 | large_intestine | MUT | 8.00 | 8.24 | insensitive |
| SK-LU-1 | lung | MUT | 8.00 | 1.54 | insensitive |
| Capan-1 | pancreas | MUT | 8.00 | 30.74 | insensitive |
| PA-TU-8902 | pancreas | MUT | 8.00 | 0.96 | insensitive |
| BEN | lung | MUT | 8.00 | 0.13 | insensitive |
| BT-474 | breast | MUT | 8.00 | -3.44 | insensitive |
| KU812 | haem._and_lymphoid_tissue | MUT | 8.00 | 19.95 | insensitive |
| HCC-15 | lung | MUT | 8.00 | -21.49 | insensitive |
| SW579 | thyroid | MUT | 8.00 | -2.22 | insensitive |
| CHL-1 | skin | MUT | 8.00 | -46.61 | insensitive |
| OV-90 | ovary | MUT | 8.00 | -1.39 | insensitive |
| HCC1954 | breast | MUT | 8.00 | -0.81 | insensitive |
| GMS-10 | central_nervous_system | MUT | 8.00 | -4.00 | insensitive |
| NCO2 | haem._and_lymphoid_tissue | MUT | 8.00 | -1.52 | insensitive |
| SK-N-FI | autonomic_ganglia | MUT | 8.00 | -7.80 | insensitive |
| B-CPAP | thyroid | MUT | 8.00 | 1.71 | insensitive |
| LU65 | lung | MUT | 8.00 | -1.36 | insensitive |
| PE/CA-PJ34  (clone C12) | upper_aerodigestive_tract | MUT | 8.00 | 1.01 | insensitive |
| LOU-NH91 | lung | MUT | 8.00 | 2.89 | insensitive |
| NCI-H2444 | lung | MUT | 8.00 | -4.64 | insensitive |
| EB2 | haem._and_lymphoid_tissue | MUT | 8.00 | -5.89 | insensitive |
| JHH-7 | liver | MUT | 8.00 | -2.28 | insensitive |
| NCI-H1651 | lung | MUT | 8.00 | -2.57 | insensitive |
| CAL-12T | lung | MUT | 8.00 | -18.61 | insensitive |
| SW 1271 | lung | MUT | 8.00 | -7.26 | insensitive |
| LCLC-103H | lung | MUT | 8.00 | -1.79 | insensitive |
| SCC-4 | upper_aerodigestive_tract | MUT | 8.00 | -11.24 | insensitive |
| 8505C | thyroid | MUT | 8.00 | -28.72 | insensitive |
| KNS-60 | central_nervous_system | MUT | 8.00 | -1.47 | insensitive |
| HLF | liver | MUT | 8.00 | -3.09 | insensitive |
| WM-983B | skin | MUT | 8.00 | -25.79 | insensitive |
| NCI-H2110 | lung | MUT | 8.00 | -4.78 | insensitive |
| LN-18 | central_nervous_system | MUT | 8.00 | -16.71 | insensitive |
| KYSE-180 | oesophagus | MUT | 8.00 | -30.58 | insensitive |
| Daoy | central_nervous_system | MUT | 8.00 | 2.94 | insensitive |
| TE-1 | oesophagus | MUT | 8.00 | -0.82 | insensitive |
| NCI-H23 | lung | MUT | 8.00 | -1.09 | insensitive |
| NCI-H1355 | lung | MUT | 8.00 | -28.98 | insensitive |
| Hs 729 | soft_tissue | MUT | 8.00 | 7.88 | insensitive |
| U-87 MG | central_nervous_system | WT | 8.00 | -45.86 | insensitive |
| JMSU-1 | urinary_tract | MUT | 8.00 | 2.12 | insensitive |
| OE33 | oesophagus | MUT | 8.00 | -2.82 | insensitive |
| TE-5 | oesophagus | MUT | 8.00 | 31.38 | insensitive |
| LK-2 | lung | MUT | 8.00 | -3.65 | insensitive |
| NCI-H1648 | lung | MUT | 8.00 | -2.43 | insensitive |
| EFE-184 | endometrium | MUT | 8.00 | -2.73 | insensitive |
| MKN74 | stomach | MUT | 8.00 | 2.29 | insensitive |
| GI-1 | central_nervous_system | MUT | 8.00 | -2.97 | insensitive |
| ChaGo-K-1 | lung | MUT | 8.00 | -18.45 | insensitive |
| KYSE-30 | oesophagus | MUT | 8.00 | -3.88 | insensitive |
| KE-39 | stomach | MUT | 8.00 | -5.22 | insensitive |
| NCI-H2009 | lung | MUT | 8.00 | -6.28 | insensitive |
| DU 145 | prostate | MUT | 8.00 | -4.91 | insensitive |
| YKG1 | central_nervous_system | MUT | 8.00 | -29.61 | insensitive |
| HMC-1-8 | breast | MUT | 8.00 | -1.67 | insensitive |
| MeWo | skin | MUT | 8.00 | 0.14 | insensitive |
| P31/FUJ | haem._and_lymphoid_tissue | MUT | 8.00 | 4.23 | insensitive |
| KYSE-70 | oesophagus | MUT | 8.00 | -0.20 | insensitive |
| BFTC-909 | kidney | MUT | 8.00 | 1.37 | insensitive |
| AN3 CA | endometrium | MUT | 8.00 | 5.06 | insensitive |
| Raji | haem._and_lymphoid_tissue | MUT | 8.00 | -0.41 | insensitive |
| P3HR-1 | haem._and_lymphoid_tissue | MUT | 8.00 | -48.91 | insensitive |
| IGROV1 | ovary | MUT | 8.00 | -1.82 | insensitive |
| FaDu | upper_aerodigestive_tract | MUT | 8.00 | 8.93 | insensitive |
| SNU-475 | liver | MUT | 8.00 | -7.74 | insensitive |
| KYSE-140 | oesophagus | MUT | 8.00 | -19.56 | insensitive |
| MOLM-16 | haem._and_lymphoid_tissue | MUT | 8.00 | 12.84 | insensitive |
| OCI-M1 | haem._and_lymphoid_tissue | MUT | 8.00 | -1.98 | insensitive |
| DMS 53 | lung | MUT | 8.00 | 9.69 | insensitive |
| HCT-15 | large_intestine | MUT | 8.00 | -20.00 | insensitive |
| PL-21 | haem._and_lymphoid_tissue | MUT | 8.00 | -37.95 | insensitive |
| CAL-78 | bone | MUT | 8.00 | -1.01 | insensitive |
| BFTC-905 | urinary_tract | MUT | 8.00 | 0.25 | insensitive |
| MEG-01 | haem._and_lymphoid_tissue | MUT | 8.00 | 0.30 | insensitive |
| KMM-1 | haem._and_lymphoid_tissue | MUT | 8.00 | -27.14 | insensitive |
| 42-MG-BA | central_nervous_system | MUT | 8.00 | -30.94 | insensitive |
| J82 | urinary_tract | MUT | 8.00 | -2.55 | insensitive |
| SU-DHL-8 | haem._and_lymphoid_tissue | MUT | 8.00 | 11.70 | insensitive |
| BT-20 | breast | MUT | 8.00 | 1.40 | insensitive |
| COLO 205 | large_intestine | MUT | 8.00 | -1.62 | insensitive |
| PK-45H | pancreas | WT | 8.00 | -18.84 | insensitive |
| EFO-21 | ovary | MUT | 8.00 | 5.85 | insensitive |
| MOG-G-UVW | central_nervous_system | WT | 8.00 | 3.49 | insensitive |
| QGP-1 | pancreas | WT | 8.00 | 6.30 | insensitive |
| HCC1806 | breast | MUT | 8.00 | 1.11 | insensitive |
| ONS-76 | central_nervous_system | WT | 8.00 | -16.12 | insensitive |
| SW 1990 | pancreas | MUT | 8.00 | -0.73 | insensitive |
| COLO 792 | skin | WT | 8.00 | 2.74 | insensitive |
| LOX IMVI | skin | WT | 8.00 | -48.25 | insensitive |
| NCI-H2452 | pleura | WT | 8.00 | -0.68 | insensitive |
| FU-OV-1 | ovary | MUT | 8.00 | -2.32 | insensitive |
| COR-L23 | lung | WT | 8.00 | -1.38 | insensitive |
| SCC-9 | upper_aerodigestive_tract | MUT | 8.00 | 10.57 | insensitive |
| DEL | haem._and_lymphoid_tissue | MUT | 8.00 | -20.11 | insensitive |
| Hs 766T | pancreas | MUT | 8.00 | -25.13 | insensitive |
| BT-549 | breast | MUT | 8.00 | -22.89 | insensitive |
| CMK-11-5 | haem._and_lymphoid_tissue | MUT | 8.00 | -25.58 | insensitive |
| NCI-H358 | lung | WT | 8.00 | -34.66 | insensitive |
| MDA-MB-453 | breast | WT | 8.00 | -3.32 | insensitive |
| SK-N-AS | autonomic_ganglia | WT | 8.00 | -3.46 | insensitive |
| HH | haem._and_lymphoid_tissue | MUT | 8.00 | 14.86 | insensitive |
| VMRC-RCZ | kidney | WT | 8.00 | 0.87 | insensitive |
| SK-OV-3 | ovary | MUT | 8.00 | 0.64 | insensitive |
| NCI-H1792 | lung | MUT | 8.00 | -2.85 | insensitive |
| NCI-H2347 | lung | WT | 8.00 | 1.66 | insensitive |
| HPB-ALL | haem._and_lymphoid_tissue | MUT | 8.00 | -2.06 | insensitive |
| HuT 78 | haem._and_lymphoid_tissue | MUT | 8.00 | 3.50 | insensitive |
| PC-3 | prostate | MUT | 8.00 | -20.98 | insensitive |
| AsPC-1 | pancreas | MUT | 8.00 | -30.80 | insensitive |
| IGR-39 | skin | MUT | 8.00 | -19.40 | insensitive |
| HD-MY-Z | haem._and_lymphoid_tissue | MUT | 8.00 | -21.14 | insensitive |
| MOR/CPR | lung | MUT | 8.00 | -1.44 | insensitive |
| COLO 741 | skin | MUT | 8.00 | 3.97 | insensitive |
| KP-3 | pancreas | MUT | 8.00 | -3.05 | insensitive |
| HSC-3 | upper_aerodigestive_tract | MUT | 8.00 | -2.32 | insensitive |
| KATO III | stomach | MUT | 8.00 | -5.58 | insensitive |
| KMS-11 | haem._and_lymphoid_tissue | WT | 8.00 | -2.60 | insensitive |
| MPP 89 | pleura | WT | 8.00 | 3.91 | insensitive |
| LC-1/sq-SF | lung | MUT | 8.00 | 12.18 | insensitive |
| KCL-22 | haem._and_lymphoid_tissue | MUT | 8.00 | -25.90 | insensitive |
| KO52 | haem._and_lymphoid_tissue | MUT | 8.00 | -34.10 | insensitive |
| MONO-MAC-1 | haem._and_lymphoid_tissue | MUT | 8.00 | -2.52 | insensitive |
| RT-112 | urinary_tract | MUT | 8.00 | 2.33 | insensitive |
| IST-MES2 | pleura | MUT | 8.00 | -3.22 | insensitive |
| BL-41 | haem._and_lymphoid_tissue | MUT | 8.00 | -3.11 | insensitive |
| KARPAS-299 | haem._and_lymphoid_tissue | MUT | 8.00 | -3.42 | insensitive |
| NCI-H2196 | lung | MUT | 8.00 | -2.21 | insensitive |
| EM-2 | haem._and_lymphoid_tissue | MUT | 8.00 | -7.05 | insensitive |
| Hs 683 | central_nervous_system | MUT | 8.00 | 3.20 | insensitive |
| NCI-H211 | lung | MUT | 8.00 | -5.53 | insensitive |
| NCI-H1581 | lung | MUT | 8.00 | 1.44 | insensitive |
| IPC-298 | skin | MUT | 8.00 | -6.63 | insensitive |
| COV362 | ovary | MUT | 8.00 | -1.49 | insensitive |
| NIH:OVCAR-3 | ovary | MUT | 8.00 | -3.87 | insensitive |
| TT2609-C02 | thyroid | MUT | 8.00 | -12.03 | insensitive |
| NB-4 | haem._and_lymphoid_tissue | MUT | 8.00 | -20.20 | insensitive |
| OPM-2 | haem._and_lymphoid_tissue | MUT | 8.00 | -2.80 | insensitive |
| KMS-26 | haem._and_lymphoid_tissue | MUT | 8.00 | 2.18 | insensitive |
| HT-29 | large_intestine | MUT | 8.00 | -14.05 | insensitive |
| LS1034 | large_intestine | MUT | 8.00 | -11.78 | insensitive |
| 5637 | urinary_tract | MUT | 8.00 | 0.50 | insensitive |
| SK-BR-3 | breast | MUT | 8.00 | 2.51 | insensitive |
| Caov-3 | ovary | MUT | 8.00 | -11.01 | insensitive |
| NCI-H747 | large_intestine | MUT | 8.00 | -4.17 | insensitive |
| Calu-3 | lung | MUT | 8.00 | -11.07 | insensitive |
| HCC70 | breast | MUT | 8.00 | -10.79 | insensitive |
| RCM-1 | large_intestine | MUT | 8.00 | -5.50 | insensitive |
| S-117 | soft_tissue | MUT | 8.00 | 0.17 | insensitive |
| SJRH30 | soft_tissue | MUT | 8.00 | -35.44 | insensitive |
| DB | haem._and_lymphoid_tissue | MUT | 8.00 | 12.10 | insensitive |
| MDA-MB-468 | breast | MUT | 8.00 | -0.27 | insensitive |
| Panc 04.03 | pancreas | MUT | 8.00 | -4.12 | insensitive |
| NCI-H810 | lung | MUT | 8.00 | -5.81 | insensitive |
| SK-MES-1 | lung | MUT | 8.00 | -5.06 | insensitive |
| RERF-GC-1B | stomach | MUT | 8.00 | -0.99 | insensitive |
| OVSAHO | ovary | MUT | 8.00 | -32.55 | insensitive |
| KNS-62 | lung | MUT | 8.00 | -16.08 | insensitive |
| NCI-H1734 | lung | MUT | 8.00 | 23.48 | insensitive |
| AU565 | breast | MUT | 8.00 | -46.46 | insensitive |
| BxPC-3 | pancreas | MUT | 8.00 | 2.85 | insensitive |
| RERF-LC-MS | lung | MUT | 8.00 | -40.30 | insensitive |
| IA-LM | lung | MUT | 8.00 | 1.08 | insensitive |
| 786-O | kidney | MUT | 8.00 | -27.93 | insensitive |
| JHH-4 | liver | MUT | 8.00 | -22.81 | insensitive |
| SK-MEL-30 | skin | MUT | 8.00 | -2.63 | insensitive |
| SH-10-TC | stomach | MUT | 8.00 | -2.17 | insensitive |
| 8-MG-BA | central_nervous_system | MUT | 8.00 | -29.68 | insensitive |
| LUDLU-1 | lung | MUT | 8.00 | -45.77 | insensitive |
| SF-295 | central_nervous_system | MUT | 8.00 | -1.59 | insensitive |
| HuCCT1 | biliary_tract | MUT | 8.00 | -1.97 | insensitive |
| SU-DHL-4 | haem._and_lymphoid_tissue | MUT | 8.00 | -29.17 | insensitive |
| NCI-H441 | lung | MUT | 8.00 | -21.27 | insensitive |
| SCC-25 | upper_aerodigestive_tract | MUT | 8.00 | -16.71 | insensitive |
| WSU-DLCL2 | haem._and_lymphoid_tissue | MUT | 8.00 | 8.42 | insensitive |
| OCI-LY10 | haem._and_lymphoid_tissue | MUT | 8.00 | -8.00 | insensitive |
| MFE-319 | endometrium | MUT | 8.00 | 1.74 | insensitive |
| MIA PaCa-2 | pancreas | MUT | 8.00 | 1.28 | insensitive |
| OC 316 | ovary | MUT | 8.00 | -2.87 | insensitive |
| BL-70 | haem._and_lymphoid_tissue | MUT | 8.00 | 5.30 | insensitive |
| DMS 114 | lung | MUT | 8.00 | 0.74 | insensitive |
| BICR 6 | upper_aerodigestive_tract | MUT | 8.00 | -6.74 | insensitive |
| MFE-296 | endometrium | MUT | 8.00 | -4.03 | insensitive |
| CAS-1 | central_nervous_system | MUT | 8.00 | -0.16 | insensitive |
| HEC-59 | endometrium | MUT | 8.00 | -1.34 | insensitive |
| PK-1 | pancreas | MUT | 8.00 | 1.38 | insensitive |
| EBC-1 | lung | MUT | 8.00 | -0.69 | insensitive |
| NCI-H1915 | lung | MUT | 8.00 | 3.71 | insensitive |
| HARA | lung | MUT | 8.00 | -1.75 | insensitive |
| SW 1783 | central_nervous_system | MUT | 8.00 | -4.15 | insensitive |
| SK-LMS-1 | soft_tissue | MUT | 8.00 | -25.67 | insensitive |
| HSC-4 | upper_aerodigestive_tract | MUT | 8.00 | -2.11 | insensitive |
| RPMI-8402 | haem._and_lymphoid_tissue | MUT | 8.00 | -4.88 | insensitive |
| Hs 578T | breast | MUT | 8.00 | 12.21 | insensitive |
| HuH-7 | liver | MUT | 8.00 | -15.66 | insensitive |
| T98G | central_nervous_system | MUT | 8.00 | -2.69 | insensitive |
| SW480 | large_intestine | MUT | 8.00 | -19.98 | insensitive |
| PC-14 | lung | MUT | 8.00 | -25.08 | insensitive |
| Hs 746T | stomach | MUT | 8.00 | 0.34 | insensitive |
| NCI-H1975 | lung | MUT | 8.00 | -20.93 | insensitive |
| Detroit 562 | upper_aerodigestive_tract | MUT | 8.00 | -3.06 | insensitive |
| NCI-H2087 | lung | MUT | 8.00 | -22.14 | insensitive |
| Calu-6 | lung | MUT | 8.00 | -24.41 | insensitive |
| SW 900 | lung | MUT | 8.00 | -4.18 | insensitive |
| NCI-H1299 | lung | WT | 8.00 | 0.83 | insensitive |
| CAMA-1 | breast | MUT | 8.00 | -26.17 | insensitive |
| COV504 | ovary | MUT | 8.00 | -21.97 | insensitive |
| OE19 | oesophagus | MUT | 8.00 | 14.48 | insensitive |
| ESS-1 | endometrium | MUT | 8.00 | 0.05 | insensitive |
| ES-2 | ovary | MUT | 8.00 | -22.65 | insensitive |
| JHOS-2 | ovary | MUT | 8.00 | -32.44 | insensitive |
| Sq-1 | lung | WT | 8.00 | 4.40 | insensitive |
| SW403 | large_intestine | MUT | 8.00 | 51.39 | insensitive |
| SCaBER | urinary_tract | MUT | 8.00 | 0.88 | insensitive |
| MHH-ES-1 | bone | MUT | 8.00 | 1.25 | insensitive |
| Hs 944.T | skin | WT | 8.00 | -32.86 | insensitive |
| WM-88 | skin | WT | 8.00 | -37.15 | insensitive |
| LN-229 | central_nervous_system | WT | 8.00 | -42.01 | insensitive |
| TE-11 | oesophagus | MUT | 8.00 | -3.43 | insensitive |
| CCK-81 | large_intestine | MUT | 8.00 | 4.13 | insensitive |
| OVCAR-8 | ovary | MUT | 8.00 | -30.79 | insensitive |
| KMRC-1 | kidney | WT | 8.00 | -42.40 | insensitive |
| NCI-H1341 | lung | WT | 8.00 | -36.36 | insensitive |
| BICR 31 | upper_aerodigestive_tract | MUT | 8.00 | -33.34 | insensitive |
| KYSE-410 | oesophagus | MUT | 8.00 | -24.33 | insensitive |
| SNU-423 | liver | MUT | 8.00 | -5.91 | insensitive |
| NCI-H1650 | lung | MUT | 8.00 | 2.72 | insensitive |
| PE/CA-PJ41  (clone D2) | upper_aerodigestive_tract | MUT | 8.00 | -0.29 | insensitive |
| PEER | haem._and_lymphoid_tissue | MUT | 8.00 | -46.94 | insensitive |
| KYSE-520 | oesophagus | MUT | 8.00 | -5.52 | insensitive |
| NCI-H1703 | lung | MUT | 8.00 | -40.27 | insensitive |
| TE-9 | oesophagus | MUT | 8.00 | -6.46 | insensitive |
| NCI-H647 | lung | MUT | 8.00 | -29.54 | insensitive |
| HSC-2 | upper_aerodigestive_tract | MUT | 8.00 | 8.13 | insensitive |
| NCI-H1693 | lung | MUT | 8.00 | -4.47 | insensitive |
| KNS-42 | central_nervous_system | MUT | 8.00 | -8.48 | insensitive |
| CAL-85-1 | breast | MUT | 8.00 | -3.57 | insensitive |
| KMS-28BM | haem._and_lymphoid_tissue | WT | 8.00 | 3.74 | insensitive |
| Capan-2 | pancreas | WT | 8.00 | -4.99 | insensitive |
| HEC-108 | endometrium | MUT | 8.00 | -7.25 | insensitive |
| KYSE-450 | oesophagus | MUT | 8.00 | -1.37 | insensitive |
| MEL-HO | skin | WT | 8.00 | -40.38 | insensitive |
| OAW42 | ovary | WT | 8.00 | -7.13 | insensitive |
| C3A | liver | WT | 8.00 | -7.36 | insensitive |
| Hs 888.T | bone | WT | 8.00 | -45.50 | insensitive |
| C32 | skin | WT | 8.00 | -3.08 | insensitive |
| Hs 618.T | lung | WT | 8.00 | -49.34 | insensitive |
| SK-MEL-24 | skin | WT | 8.00 | -47.55 | insensitive |
| SK-MEL-5 | skin | WT | 8.00 | -47.83 | insensitive |
| SW 780 | urinary_tract | WT | 8.00 | -38.52 | insensitive |
| HEC-265 | endometrium | WT | 8.00 | -37.19 | insensitive |
| UM-UC-3 | urinary_tract | MUT | 8.00 | -27.23 | insensitive |
| KYSE-510 | oesophagus | MUT | 8.00 | 3.18 | insensitive |
| Malme-3M | skin | WT | 8.00 | -39.40 | insensitive |
| NCI-H460 | lung | WT | 8.00 | 0.31 | insensitive |
| SNG-M | endometrium | WT | 8.00 | -25.75 | insensitive |
| IM95 | stomach | WT | 8.00 | 33.79 | insensitive |
| KMS-27 | haem._and_lymphoid_tissue | WT | 8.00 | -1.14 | insensitive |
| A172 | central_nervous_system | WT | 8.00 | -49.68 | insensitive |
| SW 1573 | lung | WT | 8.00 | -48.52 | insensitive |
| L-428 | haem._and_lymphoid_tissue | MUT | 8.00 | -26.31 | insensitive |
| SK-HEP-1 | liver | WT | 8.00 | -41.72 | insensitive |

IC_50_ and maximal effect (Amax) for NVP-CFC218 in each cell line are shown in µM and in percentages, respectively. Sensitivity call for each compound was applied according to a cut-off of 4 µM for IC_50_ and ‑50% for Amax. The four p53^MUT^ cell lines found to be sensitive to NVP-CFC218 are highlighted in bold.
