# Supplementary material for: A distinct p53 target gene set predicts for response to the selective p53–HDM2 inhibitor NVP-CGM097
Source: eLife. 2015 May 12;4:e06498. doi: 10.7554/eLife.06498 (PMC4468608; doi:10.7554/eLife.06498)
Supplement: Figure 2—source data 1. — DOI: http://dx.doi.org/10.7554/eLife.06498.006 [file elife-06498-fig2-data1.docx]

**Figure 2-source data 1. Classifier performance with increasing feature set size**

| Number of features | Accuracy (Mean ± SD) | Sensitivity (Mean ± SD) | Specificity (Mean ± SD) |
| --- | --- | --- | --- |
| 2 | 0.872 ± 0.031 | 0.577 ± 0.130 | 0.938 ± 0.030 |
| 5 | 0.913 ± 0.024 | 0.800 ± 0.097 | 0.938 ± 0.025 |
| 10 | 0.920 ± 0.027 | 0.860 ± 0.116 | 0.934 ± 0.030 |
| **13** | **0.928 ± 0.023** | **0.873 ± 0.104** | **0.940 ± 0.028** |
| 17 | 0.931 ± 0.021 | 0.843 ± 0.097 | 0.951 ± 0.024 |
| 20 | 0.923 ± 0.025 | 0.867 ± 0.087 | 0.936 ± 0.029 |
| 25 | 0.891 ± 0.041 | 0.780 ± 0.117 | 0.915 ± 0.037 |
| 30 | 0.892 ± 0.055 | 0.783 ± 0.128 | 0.915 ± 0.051 |
| 35 | 0.884 ± 0.043 | 0.753 ± 0.106 | 0.913 ± 0.045 |
| 40 | 0.853 ± 0.066 | 0.780 ± 0.087 | 0.869 ± 0.079 |
| 50 | 0.866 ± 0.046 | 0.817 ± 0.057 | 0.876 ± 0.058 |
| 75 | 0.808 ± 0.054 | 0.727 ± 0.101 | 0.826 ± 0.062 |
| 100 | 0.762 ± 0.062 | 0.747 ± 0.125 | 0.802 ± 0.073 |

The performance of the 13-gene predictive model is highlighted in bold.
