# Supplementary material for: A distinct p53 target gene set predicts for response to the selective p53–HDM2 inhibitor NVP-CGM097
Source: eLife. 2015 May 12;4:e06498. doi: 10.7554/eLife.06498 (PMC4468608; doi:10.7554/eLife.06498)
Supplement: Figure 3—source data 1. — DOI: http://dx.doi.org/10.7554/eLife.06498.011 [file elife-06498-fig3-data1.docx]

**Figure 3-source data 1. Sensitivity prediction and sensitivity to NVP-CFC218 and NVP-CGM097 of an external set of cell lines (n=52)**

| Cell Line Name | Lineage | Sensitivity Prediction | NVP-CGM097 IC_50_ (µM) | NVP-CFC218 IC_50_ (µM) | NVP-CGM097 sensitivity call | NVP-CFC218 sensitivity call |
| --- | --- | --- | --- | --- | --- | --- |
| ***Insensitive p53^MUT^ cells (n=10):*** | |  |  |  |  |  |
| COLO-783 | skin | sensitive | >10 | >10 | insensitive | insensitive |
| COLO-818 | skin | insensitive | >10 | >10 | insensitive | insensitive |
| VMRC-RCW | kidney | insensitive | >10 | ND | insensitive | ND |
| EFM-192A | breast | insensitive | >10 | 7.43 | insensitive | insensitive |
| IGR-37 | skin | insensitive | >10 | >10 | insensitive | insensitive |
| JHH-5 | liver | insensitive | 8.788 | 9.012 | insensitive | insensitive |
| GA-10 | haem._and_lymphoid_tissue | insensitive | 8.061 | 8.294 | insensitive | insensitive |
| HCC202 | breast | insensitive | 7.386 | 7.386 | insensitive | insensitive |
| KASUMI-1 | haem._and_lymphoid_tissue | insensitive | 6.271 | 7.137 | insensitive | insensitive |
| NCI-H1568 | lung | insensitive | 6.202 | ND | insensitive | ND |
| ***Insensitive p53^WT^ cells (n=15):*** | |  |  |  |  |  |
| Hs 688(A).T | skin | sensitive | >10 | >10 | insensitive | insensitive |
| DAN-G | pancreas | insensitive | >10 | 9.734 | insensitive | insensitive |
| Hs 834.T | skin | sensitive | >10 | >10 | insensitive | insensitive |
| DM-3 | pleura | sensitive | >10 | ND | insensitive | ND |
| HDLM-2 | haem._and_lymphoid_tissue | insensitive | >10 | 9.059 | insensitive | insensitive |
| OS-RC-2 | kidney | sensitive | >10 | ND | insensitive | ND |
| RERF-LC-KJ | lung | insensitive | 8.753 | >10 | insensitive | insensitive |
| HCC-95 | lung | insensitive | 7.129 | ND | insensitive | ND |
| IGR-1 | skin | sensitive | 6.995 | 3.669 | insensitive | insensitive |
| A101D | skin | sensitive | 6.729 | 7.784 | insensitive | insensitive |
| KMRC-2 | kidney | sensitive | 5.239 | ND | insensitive | ND |
| Caki-1 | kidney | sensitive | 4.721 | ND | insensitive | ND |
| **COLO 829** | **skin** | **sensitive** | **4.238** | **2.176** | **insensitive** | **sensitive** |
| IST-MES1 | pleura | sensitive | 4.063 | ND | insensitive | ND |
| A-498 | kidney | sensitive | 3.943 | ND | insensitive | ND |
| ***Sensitive p53^WT^ cells (n=27):*** | |  |  |  |  |  |
| NCI-H28 | pleura | insensitive | 2.827 | ND | sensitive | ND |
| COLO-849 | skin | sensitive | 2.729 | 2.934 | sensitive | sensitive |
| UACC-257 | skin | sensitive | 2.143 | 2.080 | sensitive | sensitive |
| SK-MEL-1 | skin | sensitive | 1.613 | 1.609 | sensitive | sensitive |
| Hs 940.T | skin | sensitive | 1.434 | 2.666 | sensitive | sensitive |
| JL-1 | pleura | sensitive | 1.421 | ND | sensitive | ND |
| SK-MEL-31 | skin | sensitive | 1.222 | 1.275 | sensitive | sensitive |
| A549 | lung | sensitive | 1.198 | ND | sensitive | ND |
| CAL-51 | breast | insensitive | 1.038 | 0.552 | sensitive | sensitive |
| JM1 | haem._and_lymphoid_tissue | insensitive | 0.997 | 1.102 | sensitive | sensitive |
| MV-4-11 | haem._and_lymphoid_tissue | sensitive | 0.880 | 0.981 | sensitive | sensitive |
| CCF-STTG1 | central_nervous_system | sensitive | 0.870 | 0.760 | sensitive | sensitive |
| MEL-JUSO | skin | sensitive | 0.686 | 0.801 | sensitive | sensitive |
| OCI-LY3 | haem._and_lymphoid_tissue | sensitive | 0.663 | 1.579 | sensitive | sensitive |
| UACC-62 | skin | sensitive | 0.626 | 0.740 | sensitive | sensitive |
| HuNS1 | haem._and_lymphoid_tissue | sensitive | 0.562 | 0.392 | sensitive | sensitive |
| CAL-54 | kidney | sensitive | 0.544 | ND | sensitive | ND |
| MSTO-211H | pleura | sensitive | 0.454 | ND | sensitive | ND |
| GDM-1 | haem._and_lymphoid_tissue | sensitive | 0.292 | 0.285 | sensitive | sensitive |
| SJSA-1 | bone | sensitive | 0.223 | 0.241 | sensitive | sensitive |
| D283 Med | central_nervous_system | sensitive | 0.192 | 0.191 | sensitive | sensitive |
| RS4;11 | haem._and_lymphoid_tissue | sensitive | 0.177 | 0.297 | sensitive | sensitive |
| SUP-B15 | haem._and_lymphoid_tissue | sensitive | 0.135 | 0.167 | sensitive | sensitive |
| MHH-CALL-4 | haem._and_lymphoid_tissue | sensitive | 0.109 | 0.079 | sensitive | sensitive |
| BV-173 | haem._and_lymphoid_tissue | sensitive | 0.085 | 0.051 | sensitive | sensitive |
| L-540 | haem._and_lymphoid_tissue | sensitive | 0.039 | 0.036 | sensitive | sensitive |
| EOL-1 | haem._and_lymphoid_tissue | sensitive | 0.002 | 0.003 | sensitive | sensitive |

IC_50_ values for both compounds are shown in µM. Sensitivity call for each compound was applied according to a cut-off of 3 µM. COLO 829 was the only cell line found differently sensitive to NVP-CFC218 and NVP-CGM097 according to the cut-off of 3 µM, and is highlighted in bold. ND, not determined.
