# Supplementary material for: A distinct p53 target gene set predicts for response to the selective p53–HDM2 inhibitor NVP-CGM097
Source: eLife. 2015 May 12;4:e06498. doi: 10.7554/eLife.06498 (PMC4468608; doi:10.7554/eLife.06498)
Supplement: Figure 6—source data 1. — DOI: http://dx.doi.org/10.7554/eLife.06498.017 [file elife-06498-fig6-data1.docx]

**Figure 6-source data 1. Sensitivity prediction and sensitivity to NVP-CGM097 of a set of *in vivo* PDX models (n=55)**

| PDX model | Lineage | TP53 Mutation Status | Sensitivity Prediction | NVP-CGM097 Maximum Effect (%) | NVP-CGM097 Sensitivity Call |
| --- | --- | --- | --- | --- | --- |
| HMEX3483 | Skin | WT | insensitive | 1150 | insensitive |
| HCOX1234 | Colon | MUT | sensitive | 946 | insensitive |
| HSAX3901 | Soft tissue | MUT | insensitive | 808 | insensitive |
| HCOX1290 | Colon | MUT | sensitive | 783 | insensitive |
| HCOX2659 | Colon | MUT | insensitive | 496 | insensitive |
| HMEX1655 | Skin | WT | insensitive | 489 | insensitive |
| CHLI037 | Liver | WT | insensitive | 444 | insensitive |
| HMEX2921 | Skin | WT | sensitive | 440 | insensitive |
| HCOX2182 | Colon | MUT | sensitive | 416 | insensitive |
| HMEX3676 | Skin | MUT | insensitive | 399 | insensitive |
| HMEX4455 | Skin | WT | sensitive | 376 | insensitive |
| HCOX1027 | Colon | MUT | insensitive | 295 | insensitive |
| CHLI033 | Liver | WT | insensitive | 243 | insensitive |
| HLIX2969 | Liver | MUT | sensitive | 222 | insensitive |
| HKIX2597 | Kidney | MUT | insensitive | 218 | insensitive |
| HLUX1869 | Lung | MUT | insensitive | 218 | insensitive |
| HLUX1726 | Lung | WT | insensitive | 200 | insensitive |
| HCOX2483 | Colon | MUT | insensitive | 198 | insensitive |
| CHLI015 | Liver | MUT | insensitive | 186 | insensitive |
| HKIX1169 | Kidney | MUT | insensitive | 149 | insensitive |
| HKIX2347 | Kidney | WT | sensitive | 144 | insensitive |
| HLUX1834 | Lung | MUT | insensitive | 129 | insensitive |
| HBRX2524 | Breast | MUT | insensitive | 118 | insensitive |
| HPAX1633 | Pancreas | MUT | insensitive | 108 | insensitive |
| CHLI017 | Liver | MUT | sensitive | 100 | insensitive |
| HPAX2026 | Pancreas | WT | insensitive | 93 | insensitive |
| HCOX1500 | Colon | MUT | insensitive | 63 | insensitive |
| CHLI002 | Liver | WT | insensitive | 60 | insensitive |
| HMEX2838 | Skin | WT | sensitive | -7 | sensitive |
| HCOX1329 | Colon | WT | sensitive | -8 | sensitive |
| HMEX2613 | Skin | WT | sensitive | -11 | sensitive |
| HMEX2306 | Skin | WT | insensitive | -16 | sensitive |
| HSAX2569 | Soft tissue | WT | sensitive | -18 | sensitive |
| HMEX3851 | Skin | MUT | insensitive | -19 | sensitive |
| CHLI029 | Liver | WT | insensitive | -24 | sensitive |
| HCOX1210 | Colon | WT | sensitive | -25 | sensitive |
| HMEX2700 | Skin | WT | sensitive | -30 | sensitive |
| HMEX3880 | Skin | WT | sensitive | -31 | sensitive |
| HMEX2992 | Skin | WT | insensitive | -32 | sensitive |
| HCOX0988 | Colon | MUT | insensitive | -34 | sensitive |
| HMEX2163 | Skin | MUT | sensitive | -34 | sensitive |
| HMEX3746 | Skin | WT | sensitive | -36 | sensitive |
| HCOX1480 | Colon | WT | insensitive | -38 | sensitive |
| HCOX2145 | Colon | WT | sensitive | -40 | sensitive |
| HCOX1173 | Colon | WT | sensitive | -41 | sensitive |
| HMEX4426 | Skin | WT | sensitive | -41 | sensitive |
| HMEX2723 | Skin | WT | sensitive | -42 | sensitive |
| HCOX1441 | Colon | MUT | insensitive | -44 | sensitive |
| HCOX1119 | Colon | WT | sensitive | -45 | sensitive |
| HCOX1055 | Colon | WT | sensitive | -56 | sensitive |
| HMEX1906 | Skin | WT | insensitive | -58 | sensitive |
| HCOX1303 | Colon | WT | sensitive | -70 | sensitive |
| HMEX2753 | Skin | WT | sensitive | -76 | sensitive |
| HSAX2655 | Soft tissue | WT | sensitive | -93 | sensitive |
| HMEX3486 | Skin | WT | sensitive | -100 | sensitive |

The sensitivity call was made at the maximum effect time-point during the treatment period. Maximal effects for NVP-CGM097 in each PDX model are shown in percentages. The cut-off used for sensitivity to NVP-CGM097 was based on RECIST adapted for full tumor volume measurement: models showing a progressive disease (>35% increase in tumor volume) were considered as insensitive to NVP-CGM097 treatment.
