# Supplementary material for: A distinct p53 target gene set predicts for response to the selective p53–HDM2 inhibitor NVP-CGM097
Source: eLife. 2015 May 12;4:e06498. doi: 10.7554/eLife.06498 (PMC4468608; doi:10.7554/eLife.06498)
Supplement: Figure 6—source data 2. — DOI: http://dx.doi.org/10.7554/eLife.06498.018 [file elife-06498-fig6-data2.docx]

**Figure 6-source data 2. Expression data of each of the 13 genes included in the gene signature in the set of *in vivo* PDX models (n=55)**

| PDX model | HDM2 | CDKN1A | ZMAT3 | DDB2 | FDXR | RPS27L | BAX | RRM2B | SESN1 | CCNG1 | XPC | TNFRSF10B | AEN |
| --- | --- | --- | --- | --- | --- | --- | --- | --- | --- | --- | --- | --- | --- |
| HMEX3483 | 5.430 | 8.175 | 7.442 | 7.376 | 5.720 | 7.037 | 7.970 | 9.506 | 5.349 | 9.841 | 7.744 | 7.077 | 6.491 |
| HCOX1234 | 5.666 | 7.813 | 5.759 | 8.483 | 6.110 | 10.106 | 7.955 | 7.990 | 9.174 | 10.262 | 6.604 | 7.638 | 7.685 |
| HSAX3901 | 5.768 | 8.863 | 8.309 | 5.622 | 4.166 | 7.449 | 7.373 | 8.312 | 7.184 | 9.293 | 7.100 | 5.948 | 6.573 |
| HCOX1290 | 6.228 | 9.536 | 7.882 | 8.097 | 6.888 | 8.966 | 8.600 | 8.127 | 7.000 | 11.041 | 7.530 | 8.003 | 6.905 |
| HCOX2659 | 5.167 | 8.439 | 6.468 | 6.603 | 5.068 | 7.220 | 6.947 | 7.720 | 8.320 | 11.007 | 7.611 | 6.856 | 7.468 |
| HMEX1655 | 5.258 | 6.057 | 6.508 | 7.211 | 6.208 | 8.130 | 6.721 | 8.034 | 6.325 | 10.076 | 6.214 | 6.269 | 6.584 |
| CHLI037 | 4.836 | 6.749 | 6.321 | 7.411 | 6.125 | 6.596 | 7.586 | 8.237 | 8.974 | 10.031 | 7.527 | 6.537 | 6.622 |
| HMEX2921 | 5.937 | 8.229 | 8.008 | 7.635 | 5.719 | 7.322 | 7.758 | 9.109 | 7.642 | 11.030 | 8.443 | 7.036 | 7.174 |
| HCOX2182 | 5.474 | 8.694 | 6.456 | 8.481 | 5.997 | 7.926 | 7.371 | 7.121 | 7.517 | 10.879 | 7.311 | 6.979 | 7.517 |
| HMEX3676 | 5.903 | 6.431 | 7.980 | 6.958 | 5.534 | 7.191 | 8.139 | 8.021 | 6.944 | 10.676 | 7.190 | 6.961 | 6.698 |
| HMEX4455 | 5.876 | 8.030 | 8.254 | 7.293 | 5.359 | 8.364 | 7.211 | 8.266 | 7.651 | 10.716 | 7.292 | 6.561 | 7.870 |
| HCOX1027 | 5.722 | 8.594 | 6.187 | 6.994 | 6.811 | 8.128 | 6.063 | 6.481 | 7.908 | 10.547 | 7.406 | 7.787 | 7.247 |
| CHLI033 | 5.047 | 6.415 | 5.886 | 6.590 | 5.873 | 7.751 | 7.907 | 8.257 | 5.381 | 9.860 | 6.950 | 6.386 | 6.665 |
| HLIX2969 | 5.655 | 10.482 | 7.703 | 8.807 | 7.115 | 8.233 | 8.242 | 8.831 | 6.638 | 10.193 | 7.908 | 7.214 | 7.619 |
| HKIX2597 | 4.955 | 7.985 | 6.212 | 6.934 | 4.720 | 5.561 | 6.073 | 8.053 | 6.784 | 9.254 | 7.363 | 6.368 | 6.477 |
| HLUX1869 | 6.060 | 7.502 | 6.395 | 6.419 | 6.078 | 6.755 | 6.530 | 8.795 | 6.219 | 10.000 | 6.188 | 5.007 | 6.363 |
| HLUX1726 | 5.699 | 7.551 | 6.614 | 8.267 | 5.586 | 6.298 | 7.735 | 7.990 | 5.787 | 10.456 | 6.928 | 6.051 | 6.056 |
| HCOX2483 | 5.316 | 7.997 | 6.754 | 6.417 | 6.004 | 7.358 | 7.427 | 6.885 | 6.176 | 10.284 | 7.005 | 5.992 | 6.682 |
| CHLI015 | 5.145 | 9.352 | 6.388 | 7.252 | 5.586 | 7.622 | 8.621 | 8.060 | 6.289 | 10.567 | 7.815 | 6.296 | 6.190 |
| HKIX1169 | 6.256 | 9.256 | 6.868 | 6.196 | 6.105 | 6.961 | 7.759 | 8.485 | 6.695 | 10.317 | 6.538 | 7.528 | 6.277 |
| HKIX2347 | 5.430 | 9.578 | 6.755 | 8.761 | 7.358 | 7.624 | 7.633 | 7.424 | 6.845 | 10.439 | 7.437 | 6.927 | 6.879 |
| HLUX1834 | 5.220 | 10.573 | 6.593 | 6.840 | 5.133 | 5.857 | 5.306 | 8.306 | 5.542 | 8.645 | 6.243 | 6.595 | 6.855 |
| HBRX2524 | 5.632 | 6.860 | 7.159 | 6.361 | 4.462 | 6.359 | 6.436 | 8.643 | 6.402 | 9.612 | 7.877 | 6.972 | 6.977 |
| HPAX1633 | 5.983 | 9.818 | 7.263 | 8.382 | 6.398 | 7.837 | 7.248 | 6.172 | 6.440 | 10.591 | 7.636 | 7.844 | 6.787 |
| CHLI017 | 5.858 | 6.462 | 7.459 | 7.554 | 6.319 | 7.903 | 8.294 | 7.502 | 6.391 | 10.241 | 9.209 | 5.763 | 6.987 |
| HPAX2026 | 4.963 | 9.614 | 7.369 | 6.462 | 4.749 | 6.851 | 6.723 | 7.311 | 7.117 | 8.201 | 6.850 | 6.976 | 6.140 |
| HCOX1500 | 4.614 | 8.232 | 5.892 | 7.525 | 6.797 | 7.705 | 6.607 | 8.197 | 8.808 | 9.951 | 6.945 | 7.570 | 6.310 |
| CHLI002 | 5.695 | 8.364 | 6.647 | 7.540 | 5.841 | 6.458 | 8.291 | 9.235 | 6.130 | 9.436 | 7.748 | 6.314 | 6.057 |
| HMEX2838 | 6.442 | 8.060 | 8.574 | 8.136 | 6.124 | 8.103 | 8.069 | 9.330 | 8.305 | 11.561 | 8.493 | 7.256 | 7.508 |
| HCOX1329 | 6.423 | 10.295 | 7.837 | 8.150 | 7.564 | 8.394 | 8.720 | 8.803 | 7.452 | 11.240 | 7.691 | 7.592 | 7.539 |
| HMEX2613 | 5.568 | 9.004 | 7.896 | 8.042 | 5.564 | 7.610 | 8.295 | 8.750 | 6.796 | 9.688 | 7.602 | 5.957 | 7.136 |
| HMEX2306 | 5.591 | 8.225 | 7.274 | 7.134 | 5.275 | 8.215 | 6.756 | 7.727 | 7.097 | 9.947 | 7.525 | 7.563 | 7.433 |
| HSAX2569 | 8.545 | 9.807 | 8.384 | 7.161 | 5.433 | 6.861 | 7.649 | 8.497 | 6.195 | 9.196 | 7.537 | 6.954 | 7.090 |
| HMEX3851 | 5.364 | 7.451 | 7.487 | 6.839 | 5.962 | 7.237 | 6.449 | 7.705 | 6.299 | 10.652 | 6.761 | 5.692 | 7.067 |
| CHLI029 | 5.557 | 7.021 | 7.315 | 7.134 | 5.687 | 7.789 | 8.200 | 7.830 | 8.011 | 11.341 | 7.950 | 6.325 | 6.730 |
| HCOX1210 | 6.358 | 10.003 | 6.878 | 8.703 | 7.708 | 7.896 | 6.789 | 8.086 | 6.879 | 9.456 | 7.654 | 7.204 | 7.291 |
| HMEX2700 | 6.360 | 8.087 | 9.284 | 7.912 | 5.948 | 8.390 | 8.671 | 8.237 | 7.730 | 10.165 | 7.863 | 6.720 | 7.575 |
| HMEX3880 | 6.443 | 9.152 | 8.727 | 8.732 | 6.740 | 7.665 | 7.757 | 9.113 | 7.827 | 11.477 | 7.731 | 6.485 | 7.759 |
| HMEX2992 | 5.501 | 7.726 | 8.460 | 7.283 | 5.956 | 7.489 | 7.678 | 8.557 | 7.144 | 9.737 | 7.537 | 5.779 | 6.654 |
| HCOX0988 | 5.198 | 9.191 | 7.281 | 7.358 | 5.839 | 7.241 | 6.951 | 8.019 | 8.302 | 10.062 | 6.936 | 6.786 | 6.575 |
| HMEX2163 | 5.838 | 8.887 | 8.465 | 9.224 | 6.382 | 9.297 | 8.510 | 8.293 | 6.681 | 9.378 | 6.970 | 7.397 | 7.900 |
| HMEX3746 | 5.914 | 8.031 | 8.359 | 7.529 | 5.642 | 8.015 | 8.047 | 8.317 | 7.600 | 10.930 | 7.256 | 7.095 | 6.937 |
| HCOX1480 | 5.473 | 6.657 | 6.138 | 6.939 | 5.700 | 7.934 | 7.771 | 7.806 | 7.298 | 10.657 | 7.338 | 6.257 | 6.776 |
| HCOX2145 | 6.047 | 10.239 | 7.712 | 8.270 | 5.980 | 7.522 | 5.860 | 7.713 | 7.015 | 10.053 | 7.602 | 8.412 | 7.117 |
| HCOX1173 | 6.066 | 8.704 | 7.550 | 8.191 | 7.469 | 8.201 | 8.104 | 8.256 | 7.404 | 11.109 | 7.602 | 7.755 | 7.026 |
| HMEX4426 | 7.939 | 7.378 | 7.620 | 6.640 | 4.539 | 6.991 | 7.813 | 7.035 | 6.857 | 11.887 | 7.127 | 6.086 | 7.955 |
| HMEX2723 | 6.442 | 9.056 | 8.543 | 8.323 | 7.247 | 8.472 | 8.194 | 8.473 | 7.189 | 9.542 | 7.224 | 7.255 | 8.322 |
| HCOX1441 | 5.152 | 9.631 | 7.812 | 7.417 | 5.272 | 6.925 | 7.221 | 7.632 | 7.523 | 10.264 | 7.090 | 7.672 | 6.442 |
| HCOX1119 | 5.983 | 10.759 | 7.167 | 7.854 | 6.730 | 8.461 | 8.308 | 8.069 | 6.656 | 10.187 | 7.311 | 8.246 | 7.393 |
| HCOX1055 | 5.700 | 9.987 | 8.494 | 8.528 | 6.505 | 8.452 | 8.341 | 7.693 | 9.042 | 11.374 | 7.713 | 8.141 | 7.446 |
| HMEX1906 | 5.190 | 6.071 | 7.418 | 6.913 | 5.751 | 7.484 | 6.351 | 7.712 | 6.213 | 10.881 | 8.051 | 6.701 | 6.753 |
| HCOX1303 | 5.418 | 9.901 | 7.652 | 7.082 | 6.819 | 8.361 | 7.147 | 7.827 | 9.504 | 10.439 | 6.621 | 7.112 | 6.844 |
| HMEX2753 | 5.949 | 10.538 | 9.378 | 8.870 | 8.254 | 8.323 | 7.431 | 8.987 | 8.619 | 10.906 | 8.694 | 6.631 | 7.622 |
| HSAX2655 | 9.443 | 8.119 | 7.338 | 7.393 | 5.854 | 7.785 | 7.909 | 8.541 | 6.443 | 11.849 | 7.760 | 6.337 | 7.216 |
| HMEX3486 | 5.765 | 8.177 | 8.788 | 7.497 | 6.037 | 7.898 | 7.485 | 9.135 | 6.876 | 9.134 | 7.855 | 6.516 | 7.093 |
